# Clinical outcome of recurrent giant cell tumor of the extremity in the era before molecular target therapy: the Japanese Musculoskeletal Oncology Group study

**DOI:** 10.1186/s12891-016-1163-z

**Published:** 2016-07-22

**Authors:** Akihiko Takeuchi, Hiroyuki Tsuchiya, Takeshi Ishii, Yoshihiro Nishida, Satoshi Abe, Akihiko Matsumine, Akira Kawai, Kenichi Yoshimura, Takafumi Ueda

**Affiliations:** Department of Orthopaedic Surgery, Kanazawa University Graduate School of Medical Science, 13-1 Takara-machi, Kanazawa, 920-8641 Japan; Department of Orthopedic Surgery, Chiba Cancer Center Hospital, 666-2 Nitona, Chuo-ku, Chiba 260-8717 Japan; Department of Orthopaedic Surgery, Nagoya University School of Medicine, 65 Tsurumai, Showa-ku, Nagoya, 466-8550 Japan; Department of Orthopaedics, Teikyo University School of Medicine, 11-1 Kago 2-Chome, Itabashi-Ku, Tokyo 173-8605 Japan; Department of Orthopaedic Surgery, Mie University Faculty of Medicine, 2-174 Edobashi, Tsu-shi, Mie 514-8507 Japan; Department of Musculoskeletal Oncology, National Cancer Center Hospital, 5-1-1 Tsukiji, Chuo-ku, Tokyo 104-0045 Japan; Innovative Clinical Research Center (iCREK), Kanazawa University Hospital, 13-1 Takara-machi, Kanazawa, 920-8641 Japan; Department of Orthopaedic Surgery, Osaka National Hospital, 2-1-14 Hoenzaka, Chuo-ku, Osaka 540-0006 Japan

**Keywords:** Giant cell tumor of bone, Local recurrence, Extremity, Surgical treatment, Multicenter study

## Abstract

**Background:**

Giant cell tumor of the bone (GCTB) is classified as an intermediate, locally aggressive but rarely metastasizing tumor. The mainstay of treatment for the treatment of GCTB had been the surgical removal until an anti- receptor activator of nuclear factor-kappa B ligands (RANKL) antibody denosumab was developed. And favorable responses and the possibility of surgical downstaging have been reported. However, the long-term outcome of denosumab has not yet been confirmed and moreover the long-term clinical outcome after the recurrence of GCTB in the era before molecular target therapy is still uncertain. The aim of this study was to evaluate the long-term clinical outcome of recurrent GCTB of the extremity in the era before molecular target therapy and to determine the factors that affect the repetitive recurrence and sacrifice of adjacent joints.

**Methods:**

This multicenter study focused only recurrent GCTB of the extremity with no medical treatment and included 103 patients treated from 1980 to 2008.

**Results:**

Thirty-two (31.1 %) patients developed repetitive recurrences after salvage surgery. Second curettage and venue of initial surgery (non-cancer hospital) were both significantly correlated with repetitive recurrence in univariate (*P* = 0.034 and *P* = 0.002) and multivariate (*P* = 0.004 and *P* = 0.001) analyses. Seventy-two (76.6 %) of 94 patients achieved successful preservation of adjacent joints. Campanacci Grade III was significantly correlated with sacrifice of the adjacent joint by univariate statistical analysis (*P* = 0.019), although its impact was only marginally significant by multivariate analysis (*P* = 0.059). Seventeen patients (16.5 %) developed distant metastasis, and one patient (0.97 %) developed malignant transformation. Finally, 94/103 patients (91.3 %) with recurrent GCTB were successfully rendered NED by further surgical treatment.

**Conclusions:**

We concluded that repetitive, thorough curettage with surgical adjuvant treatment resulted in a favorable rate of adjacent joint preservation (76.6 %), but recurettage inferred a risk of repetitive recurrences. Although the treatment strategy against the recurrent GCTB is being updated with denosumab, we believe that our data will be useful for future comparisons with the long-term clinical benefit of denosumab.

## Background

Giant cell tumor of the bone (GCTB) is classified as an intermediate, locally aggressive but rarely metastasizing tumor. It accounts for 4–5 % of primary bone tumor and 20 % of all benign tumors [1]. The mainstay of treatment for the treatment of GCTB had been the surgical removal. To reduce local recurrence, a variety of adjuvant treatments using phenol, liquid nitrogen, high-speed burr, or methylmethacrylate cement have been established [2–4]. The advantage of these adjuvant treatments in the treatment of GCTB has generally been accepted in the field. It has been reported that adjuvant treatment contributes to better prevention of local recurrence (0–34 %) [4, 5] than treatment without adjuvants (12–47 %) [5–8] GCTB expresses receptor activator of nuclear factor-kappa B (RANK) and stromal cells that express RANK ligands (RANKL) [9]. Therefore, an anti-RANKL antibody denosumab was developed for the treatment of GCTB [10]. The favorable responses and the possibility of surgical downstaging were reported [11, 12]. Although denosumab was predicted to reduce osteolysis and control disease progression in patients with GCTB, the long-term outcome of denosumab has not yet been confirmed. On the other hand, there is little data of the long-term clinical outcome of GCTB after recurrence including the rate of sacrificing adjacent joint due to the additional surgical treatment. The purpose of this study was to evaluate the long-term clinical outcome of recurrent GCTBs of the extremity in the era before molecular target therapy and to determine the factors that influence repetitive recurrence and sacrifice of adjacent joint.

## Methods

Data regarding age, gender, location, Campanacci grade [5], initial treatment, venue of initial treatment, time to local recurrence, treatment of recurrence, number of recurrences, distant metastasis, malignant transformation, term of follow-up, and outcome were collected by questionnaire from the 20 cancer centers and university hospitals that participate in the Japanese Musculoskeletal Oncology Group (JMOG) network. Campanacci grade is based on the radiographic appearance. Grade I tumor is associated with a well-marginated border of a thin rim of mature bone and the cortex is intact or slightly thinned but not deformed. Grade II tumor appears relatively well-defined margins but no radiopaque rim. Grade III tumors has fuzzy borders [5]. The inclusion criterion was histologically proven recurrent GCTB in the extremities with no medical treatment. A total of 138 patients were treated for recurrent GCTB from 1980 to 2008. We excluded cases with tumors at axial sites (*N* = 4) and cases of recurrence in soft tissue (*N* = 6). We excluded another 25 patients because of lack of information with regards to Campanacci grade. Therefore, we reviewed 103 patients in this study.

We divided the patients into two groups depending on the anatomical site of the disease to evaluate the risk of sacrificing the major joints: site A included the distal radius, proximal humerus, proximal femur, distal femur, proximal tibia, and distal tibia and site B included the ulna, fibula, and talus (Table 1). Site A is adjacent to large joints, including the wrist, shoulder, hip, knee, and ankle which cause severe loss of limb function after being sacrificed, whereas site B includes all other small joints [13].

**Table 1:** Distribution of anatomical sites (*N* = 103)

	No. of cases	Percentage
Site A		
Humerus/proximal	6	5.8
Radius/distal	12	11.7
Femur/proximal	9	8.7
Femur/distal	28	27.2
Tibia/proximal	37	35.9
Tibia/distal	3	2.9
Site B		
Ulna/distal	3	2.9
Fibula/proximal	1	1
Hand	2	1.9
Foot	1	1
Patella	1	1

Disease-free survival after the second surgery was defined as the time interval from the second surgery to the second recurrence as analyzed using the Kaplan–Meier method, and the factors influencing repetitive recurrence (more than two recurrences) were determined by uni- and multivariate analyses. The chi-square test and logistic analysis were used to determine the factors influencing the preservation of adjacent joint (site A).

Statistical significance was defined by probability (*P*) values ≤ 0.05. Data were analyzed with Statistical Package for Social Sciences (SPSS) for Windows (version 19; SPSS Inc., Chicago, IL, USA).

## Results

The study included 53 males and 50 females, with a median age of 34 years (range, 12–82 years), and the median follow-up period was 114 months (range, 11–408 months). Anatomical locations included the upper extremity in 23 patients and the lower extremity in 80 patients (Table 1). Ninety-one patients underwent primary treatment at our group’s institution (Group P). Twelve patients were referred to our group’s institution with recurrence of GCTB after treatment elsewhere (Group R).

**Fig. 1:**
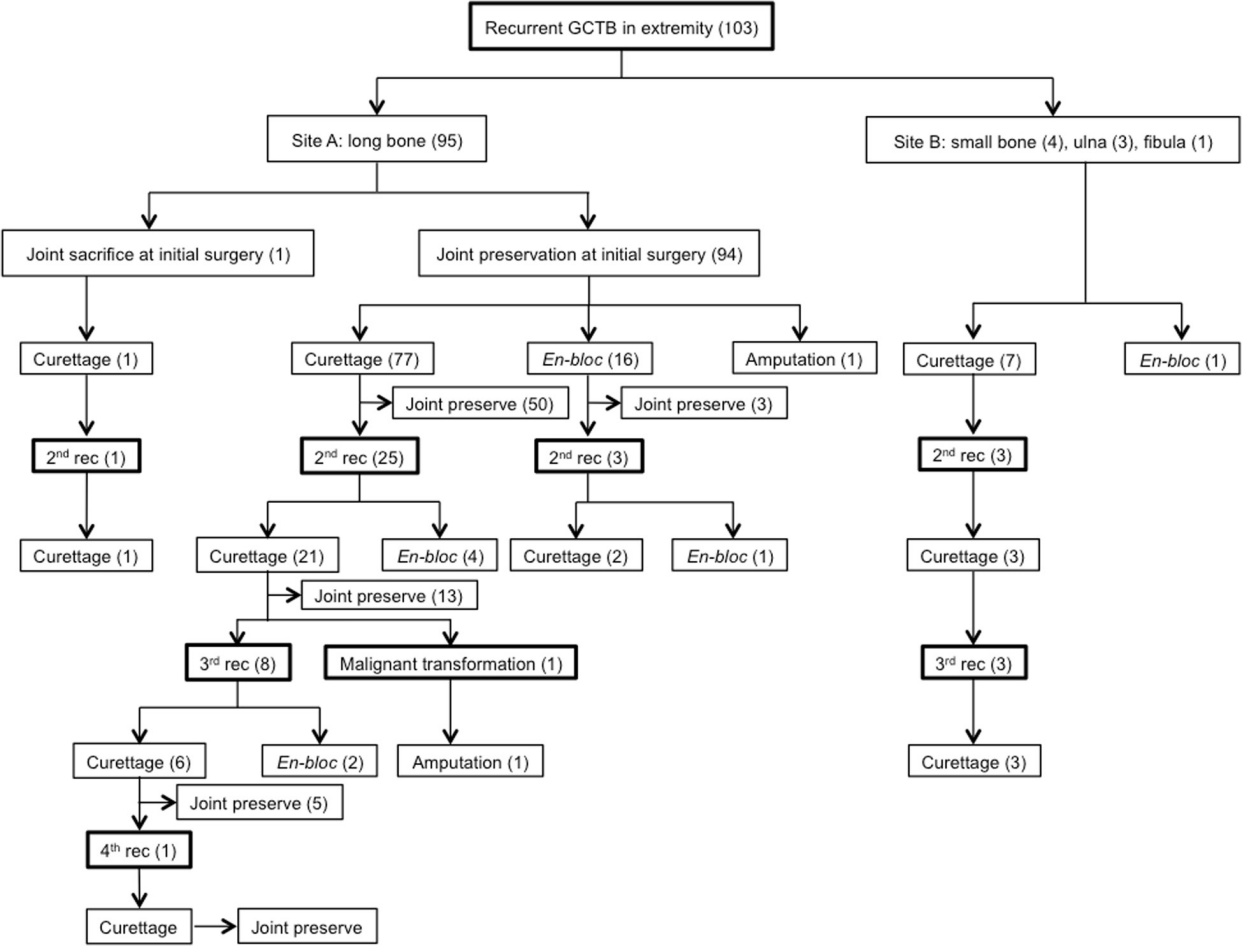
Flowchart of treatment course. (Rec, recurrence; *En-bloc*, *En-bloc* excision)

Ninety-five patients were included in site A and 8 patients in site B (Table 1). The second surgery included curettage in 85, en-bloc excision in 17, and amputation in one patient (Fig. 1). Thirty-two patients developed a second recurrence (29 patients in site A and three patients in site B). The third surgery included curettage in twenty-seven patients, and en-bloc excision in five patients. Eleven patients developed a third recurrence (eight patients in site A and three patients in site B). The forth surgery included curettage in nine patients, and en-bloc excision in two patients. One patient developed malignant transformation after the third surgery, and therefore, amputation was performed. One patient developed a forth recurrence and was treated by recurettage. Factors influencing re-recurrence-free survival as identified by univariate analysis included the venue of primary treatment and procedure of second surgery (Table 2). Multivariate analysis revealed that age (≥35 y.o., *P* = 0.006), Group R (*P* < 0.001) and second curettage (*P* = 0.004) were independent predictors of worse re-recurrence-free survival (Table 2).

**Table 2:** Factor analysis for repetitive recurrence

			Univariate analysis	Cox proportional hazards regression analysis				
Factors	No. of patients (event)	5-y re-recurrent free survival	*P* value	Wald Statistic	Regression coefficient (B)	Relative risk (eB)	95 % CI	*P* value
Age								
< 35 y.o.	66 (18)	0.626	0.332					
≥ 35 y.o.	37 (14)	0.594		7.526	1.119	3.062	1.377–6.811	0.006
Gender								
Male	53 (13)	0.656	0.341					
Female	50 (19)	0.581						
Tumor site								
Upper extremity	79 (23)	0.591	0.089					
Lower extremity	24 (9)	0.636						
Tumor location								
Site A	95 (29)	0.616	0.557					
Site B	8 (3)	0.625						
Campanacci								
I	12 (4)	0.550	0.433					
II	71 (19)	0.657						
III	20 (9)	0.524						
Initial procedure								
Curettage	98 (30)	0.603	0.785					
*En bloc* excision	5 (2)	0.800						
Status								
Group P	91 (24)	0.682	0.002					
Group R	12 (8)	0.563		10.831	1.625	5.078	1.929–13.363	< 0.001
Second surgery								
Curettage	84 (29)	0.541	0.034	8.522	2.054	8.522	1.964–30.590	0.004
*En-bloc* excision	19 (3)	0.872						

Next, we examined the factors influencing sacrifice of the adjacent joint in the 94 patients in site A. One patient in site A had his adjacent joint sacrificed at the time of initial surgery; therefore, we excluded this patient from the analysis. Fifty-three of 94 patients achieved preservation of the adjacent joint after the second surgery. To this end, 13/25 and 5/8 patients achieved preservation of the adjacent joint after the third and fourth surgery, respectively (Fig. 1). Hence, successful preservation of the adjacent joint was achieved in 72/94 patients (76.6 %). Factors influencing sacrifice of the adjacent joint as identified by chi-square analysis included Group R (*P* = 0.036) and Campanacci grade III (*P* = 0.019) (Table 3). Multivariate analysis revealed Campanacci grade III (*P* = 0.059) as a marginally significant predictor of adjacent joint sacrifice (Table 3). In this study, the highest rate of joint sacrifice was observed for the proximal femur (55.6 %), whereas the rate of the other joints ranged from 0 to 28.6 % (Table 4).

**Table 3:** Analysis for joint preservation

*N* = 95		Joint status after final surgery		Chi-square analysis		Logistic analysis			
		Preserve	Sacrifice	*P* value	*X* ^2^ value	Standardized coefficient (B)	Relative risk (e^B^)	95 % CI	*P* value
Age grade	≤ 35	50	12						
	> 35	22	10	0.197	1.666	0.934	2.546	0.853–7.600	0.094
Gender	F	37	8						
	M	35	14	0.217	1.524	0.575	1.777	0.606–5.209	0.295
Patient status	Group P	67	17						
	Group R	5	5	0.036	4.415	0.987	2.683	0.548–13.139	0.223
Campanacci Grade	I or II	62	14						
	III	10	8	0.019	5.498	1.172	3.230	0.957–10.903	0.059
Recurrence type	Repetitive	19	9	0.0192	1.699	0.475	0.622	0.200–1.930	0.411
	Single	53	13

**Table 4:** Joint status after final surgery by location and Campanacci grade

Location (number and rate of joint sacrificing)	Campanacci grade	Joint status after final surgery	
		Preserve	Sacrifice
Humerus/Proximal (*N* = 6, 16.7 %)	II	5	1
Radius/Distal (*N* = 12, 16.7 %)	II	9	0
	III	1	2
Femur/ Proximal (*N* = 9, 55.6 %)	II	3	5
	III	1	0
Femur/Distal (*N* = 28, 28.6 %)	I	6	0
	II	9	5
	III	5	3
Tibia/Proximal (*N* = 37, 18.9 %)	I	1	1
	II	26	3
	III	3	3
Tibia/Distal (*N* = 3, 0 %)	I	1	0
	II	2	0

The number of patients who developed distant metastasis in the absence of malignancy was 17 (16.5 %; lung metastasis in 15, bone metastasis in one, and both types of metastasis in one patient). Nine patients received pulmonary metastasectomy, and the disease was conservatively controlled in eight patients; all are still alive after a mean follow-up period of 78 months, with 10 patients having no evidence of the disease (NED), six patients are alive with the disease (AWD), and one patient has died because of the disease (DOD). The final status of the patients is as follows: NED, 94 patients; AWD, 8 patients; DOD, 1 patient.

## Discussion

In this study, we found that repetitive recurrence was not a risk factor for sacrifice of the adjacent joint (wrist, shoulder, hip, knee, and ankle) and the adjacent joint was still preserved in 76.6 % of the patients who had received an initial surgery with curettage. Initial treatment venue (Group R) and Campanacci Grade III were both risk factors for sacrificing the adjacent joint by univariate analysis, although the impact of Campanacci Grade III was only marginally significant by multivariate analysis.

Prior to the introduction of denosumab, the clinical outcome of treatment for recurrent GCTB has been reported in several papers. Prosser et al. [8] performed repeat curettage after local recurrence of GCTB in 43 cases, with a success rate of 100 % in patients who previously had curettage and 79.3 % in patients referred from elsewhere. McGough et al. [14] treated 45 cases of recurrent GCTB and found that incomplete initial surgery, a delay of more than 6 months in the diagnosis of recurrence, and subchondral recurrence of the tumor were factors contributing to the failure to salvage the joint.

We previously reported the clinical outcome of recurrent GCTB in the extremities treated by the Eastern Asian Musculoskeletal Oncology Group (EAMOG). We retrospectively reviewed 110 patients and analyzed the factors influencing the number of recurrences and distant metastasis. In that study, 98/110 patients (89.1 %) were successfully rendered NED by further treatment. The adjacent joint (wrist, shoulder, hip, knee, and ankle) was still preserved in 48.5 % of the patients who had received initial surgery with curettage. The initial treatment venue (Group R) was a risk factor for sacrificing the adjacent joint [15].

The site of the tumor, particularly the proximal femur, may be a factor influencing the risk of sacrificing the adjacent joint. Errani et al. [16] reported a high recurrence rate in the proximal femur compared with that in other sites. They also mentioned that special attention must be given to the proximal femur because of the difficulties in treating it. In the present study, 22/94 patients eventually required amputation, prosthetic replacement, or arthrodesis. Although the indications of these procedures were not standardized across all centers, the venue of the initial treatment (Group R) (*P* = 0.036) and Campanacci grade III (*P* = 0.019) were both risk factors for adjacent joint sacrifice in univariate analysis. The repeated curettage was related to repetitive recurrence; however, repetitive recurrence was not associated with sacrifice of adjacent joints. The primary surgery with careful follow-up is critical in preventing resection of the adjacent joint and maintaining the function of the joint. Treatment for recurrent GCTB of the extremities should aim to preserve the function of the adjacent joint by meticulous curettage with adjuvant treatment.

It this study, the incidence of lung metastasis appears to be high (14.6 %) compared with the previously reported incidence in the general GCTB population (approximately 3 %) [8, 17]. Although the cohort of patients in this study included only those with recurrent GCTB which may represent a selection bias towards patients with a higher risk of developing metastasis, there are several papers reported the relationship between local recurrence and metastasis [16–19].

Most cases of malignant transformation of GCTB occur after radiation treatment, and high-grade malignant transformation in the absence of previous irradiation is very rare [20]. Bertoni et al. [21] reported six cases of postsurgical secondary malignant GCTB without prior irradiation. They also mentioned that the average interval between the diagnosis of benign GCTB and that of sarcoma of these patients was 18 years, which is much longer than the average interval observed in patients who receive previous radiotherapy (9 years). In this study, one patient (0.97 %) developed malignant transformation without prior irradiation.

With GCTBs expressing RANK and stromal cells expressing RANKL [9], the anti-RANKL antibody (denosumab) was introduced for the treatment of GCTB [10], and favorable responses were reported [11, 12]. Rutkowskiet et al. [12] reported that among patients with resectable GCTB treated with denosumab and for whom curative intent surgery was planned and believed to be associated with significant morbidity before enrollment, 48 % had not yet undergone surgery altogether and remained on monthly denosumab treatments at the time of the data cutoff. Moreover, another 38 % patients were treated with denosumab and underwent a less invasive surgical procedure than was planned at the time of entering the study. Denosumab is predicted not only to the reduce osteolysis and control disease progression in patients with GCTB but also to ameliorate the clinical outcome of recurrent GCTB. However, further studies are still warranted to determine the long-term outcome of denosumab. This study will be useful for future studies evaluating the long-term clinical benefit of denosumab.

A limitation of this study is that it was a multicenter retrospective study with no randomization protocol of surgical procedures. Thus, the indication of the surgery (recurettage or *en bloc* excision) was not identical across the centers. The final decision of the type of surgery was made by the operating surgeon in each institution. Although the total number of patients was relatively large for recurrent GCTB, the sample size in each joint was relatively small to draw conclusions. In cox proportional hazards regression analysis, 32 events (repetitive recurrence) provided a 80 % power to detect a relative risk of 2.7 in re-recurrence-free survival.

## Conclusions

In this cooperative study, recurettage inferred a risk of repetitive recurrences but not of having the adjacent joint sacrificed. Repetitive, thorough curettage with surgical adjuvant treatment resulted in a favorable rate of adjacent joint preservation. Although the treatment strategy against the recurrent GCTB will be changed with denosumab, we believe that our data will be useful for future comparisons with the long-term clinical benefit of denosumab.

## Abbreviations

AWD, alive with the disease; DOD, died because of the disease; GCTB, giant cell tumor of bone; JMOG, Japanese Musculoskeletal Oncology Group; NED, no evidence of the disease; RANK, receptor activator of nuclear factor-kappa B; RANKL, receptor activator of nuclear factor-kappa B ligands
